# Health Tract, No. 163

**Published:** 1865

**Authors:** 


HEALTH TRACT, No. 163.

### FIFTH AVENUE SIGHTS.

A streak of white petticoat is one of the most refreshing sights to be met with on the great thoroughfare of fashion, folly, and snobbery, because it is a pretty sure index that the wearer of the same possesses those characteristics of the sex, which make of a true woman the priceless being that she is; and first of all, that personal purity, which emanates from a pure heart and an exalted nature. When, in the fashion of the times, a woman shows her petticoat on the street, it indicates the possession of force of character, of independence of thought, and a consciousness of tidiness, which of itself extorts our admiration and commands respect.

In our morning walk down-town the other day, we noticed that some careless footman had trod on an immense green worm and smashed it all abroad into a jelly; then there was a demonstration that some hound or whelp or cur of low degree, had taken an emetic; and a little farther on, that same or other, with the beef cattle which are every morning driven along the avenue, and for some reason best known to themselves, prefer the sidewalk, had made other unseemly exhibitions; while almost everywhere was seen the foul tobacco-spit of some human beast; or the product of a consumptive cough; or a blow from a nose, not emptied before within twelve hours. In the course of an hour or two, the "prime part" of all these abominations is deposited on the velvet carpets which spread the parlors of the regal mansions lining the magnificent thoroughfare, by means of the trailing dresses, which senseless and inexorable fashion demands of her idiotic votaries. A part of the above filth is flapped by the dress against the stockings, gaiters, and petticoat of the wearer, the fumes of the detestable compounds rising upward about the person, saturating the clothing, and making the individual, however magnificently dressed, really unfit to be approached with a forty-foot pole.

Our ladies will take a bran new dress of the most faultless figure and most costly material, and walk the streets with its first wearing; the inner edge of the lower portion of the dress trails on the pavement, and in an hour is irretrievably stained and soiled, and begrimed; it may be only the lining; but it dries on it, and there remains, unless the ladies renew the lining at every wearing; but whose cheek would not mantle with shame, if this same portion of the dress was stretched out for exhibition? A true woman abhors dry dirt as much as dirt that is wet; and would feel a conscious degradation if she knew the inside of an otherwise faultlessly clean dress was soiled, while she makes it of more account to have her inner garments, and the most undermost clothing the sweeter and the cleaner the nearer they approach her person. Holding up the dress, and displaying a snow-white petticoat, prevents all this; and tells plainly that the wearer is a true woman; tidy, pure, and independent in thought. But not one in a hundred has force of character, or thoroughness enough to raise the dress, even in the few cases where it is attempted; proving clearly that most of the women who promenade the Avenue are slovens, or are among the new rich, and are conscious that the proper holding up of the dress would demonstrate their plebeian origin, in the thick ankle and the immense flat foot.

